# A Pattern Categorization of CT Findings to Predict Outcome of COVID-19 Pneumonia

**DOI:** 10.3389/fpubh.2020.567672

**Published:** 2020-09-18

**Authors:** Chao Jin, Cong Tian, Yan Wang, Carol C. Wu, Huifang Zhao, Ting Liang, Zhe Liu, Zhijie Jian, Runqing Li, Zekun Wang, Fen Li, Jie Zhou, Shubo Cai, Yang Liu, Hao Li, Zhongyi Li, Yukun Liang, Heping Zhou, Xibin Wang, Zhuanqin Ren, Jian Yang

**Affiliations:** ^1^Department of Radiology, The First Affiliated Hospital of Xi'an Jiaotong University, Xi'an, China; ^2^Department of Thoracic Imaging, University of Texas M.D. Anderson Cancer Center, Houston, TX, United States; ^3^Department of Radiology, The Eighth Hospital of Xi'an, Xi'an, China; ^4^Department of Radiology, Xi'an Chest Hospital, Xi'an, China; ^5^Department of Cardiology, The First Affiliated Hospital of Xi'an Jiaotong University, Xi'an, China; ^6^Department of Critical Care Medicine, The First Affiliated Hospital of Xi'an Jiaotong University, Xi'an, China; ^7^Department of Critical Care Medicine, Wuhan No.9 Hospital, Wuhan, China; ^8^Department of Radiology, Ankang Center Hospital, Ankang, China; ^9^Department of Radiology, Hanzhong Center Hospital, Hanzhong, China; ^10^Department of Radiology, Baoji Center Hospital, Baoji, China

**Keywords:** novel coronavirus disease, computed tomography, CT pattern, clinical outcome, pulmonary sequelae

## Abstract

**Background:** As global healthcare system is overwhelmed by novel coronavirus disease (COVID-19), early identification of risks of adverse outcomes becomes the key to optimize management and improve survival. This study aimed to provide a CT-based pattern categorization to predict outcome of COVID-19 pneumonia.

**Methods:** One hundred and sixty-five patients with COVID-19 (91 men, 4–89 years) underwent chest CT were retrospectively enrolled. CT findings were categorized as Pattern 0 (negative), Pattern 1 (bronchopneumonia pattern), Pattern 2 (organizing pneumonia pattern), Pattern 3 (progressive organizing pneumonia pattern), and Pattern 4 (diffuse alveolar damage pattern). Clinical findings were compared across different categories. Time-dependent progression of CT patterns and correlations with clinical outcomes, i.e.„ discharge or adverse outcome (admission to ICU, requiring mechanical ventilation, or death), with pulmonary sequelae (complete absorption or residuals) on CT after discharge were analyzed.

**Results:** Of 94 patients with outcome, 81 (86.2%) were discharged, 3 (3.2%) were admitted to ICU, 4 (4.3%) required mechanical ventilation, 6 (6.4%) died. 31 (38.3%) had complete absorption at median day 37 after symptom onset. Significant differences between pattern-categories were found in age, disease severity, comorbidity and laboratory results (all *P* < 0.05). Remarkable evolution was observed in Pattern 0–2 and Pattern 3–4 within 3 and 2 weeks after symptom-onset, respectively; most of patterns remained thereafter. After controlling for age, CT pattern significantly correlated with adverse outcomes [Pattern 4 vs. Pattern 0–3 [reference]; hazard-ratio [95% CI], 18.90 [1.91–186.60], *P* = 0.012]. CT pattern [Pattern 3–4 vs. Pattern 0–2 [reference]; 0.26 [0.08–0.88], *P* = 0.030] and C-reactive protein [>10 vs. ≤ 10 mg/L [reference]; 0.31 [0.13–0.72], *P* = 0.006] were risk factors associated with pulmonary residuals.

**Conclusion:** CT pattern categorization allied with clinical characteristics within 2 weeks after symptom onset would facilitate early prognostic stratification in COVID-19 pneumonia.

## Introduction

Since the latter part of December of 2019, an outbreak of respiratory disease caused by severe acute respiratory syndrome-coronavirus-2 (SARS-CoV-2) has become a pandemic (1). As of May 29, 2020, 5,704,736 laboratory-confirmed cases and 357,736 deaths have been reported (2). Numerous studies have revealed the epidemiological, clinical, and radiological characteristics of the novel coronavirus disease (COVID-19) (3–6). Despite the fact that more than 80% of infected patients manifest with only mild clinical symptoms (3), early identifying the risks of an adverse outcome remains the key to optimize management and improve survival. Previous studies found that advanced age and presence of comorbidity (e.g., cardiovascular disease or hypertension) were risk factors associated with an adverse outcome such as admission to intensive care unit (ICU), need for mechanical ventilation, or death (7, 8). In addition, some laboratory indicators e.g., elevated hypersensitive troponin I, leukocytosis, neutrophilia, lymphopenia, and elevated D-dimer were found to be linked with unfavorable clinical outcomes (7–9). Presence of consolidation on computed tomography (CT) was also considered to be predictive of poor outcome in COVID-19 (10). Despite the above, the identification of early prognostic signs of COVID-19 remains of urgent importance due to the diversity in clinical and imaging findings as well as the severity and rapid progression of disease.

It is recognized that CT plays a central role in diagnosis and management of COVID-19 pneumonia (11–13). Reported CT findings of COVID-19 pneumonia included the ground glass opacities (GGO), consolidation, septal thickening mainly along the subpleural lungs or bronchovascular bundles or diffusely in the entire lungs (14). These are highly suggestive of lung organization response to injury from COVID-19 pneumonia, similar to radiological findings in the diffuse alveolar damage (DAD) and organizing pneumonia (OP) (15). Pathological studies also observed DAD in patients who succumbed to COVID-19 (16). Previous studies have demonstrated a decreased survival rate of 35–50% in DAD, while most patients with OP had better prognosis (15). In this regard, a pattern categorization of COVID-19 pneumonia, i.e., DAD and OP patterns may help the prognostic stratification. Based on the prior study regarding influenza A (H1N1) pneumonia (17), Lee also suggested a pattern categorization of COVID-19, i.e., bronchopneumonia, OP and DAD (18). A rapid progression of OP-like injury in Severe Acute Respiratory Syndrome (SARS) was considered to be predictive of a protracted clinical course (19). This may suggest a progressive subtype of OP pattern. Based on the aforementioned knowledge, a CT pattern categorization of COVID-19 pneumonia, i.e., bronchopneumonia, OP, progressive OP and DAD may have potential prognostic implications, e.g., adverse outcome, clinical course with recovery. As healthcare systems in many countries are overwhelmed with COVID-19 patients, improved prediction of the course of the disease based on early findings can assist with improved utilization of limited resources. To this end, this study aimed to investigate the prognostic significance of a CT pattern categorization in conjunction with the clinical indicators on clinical outcome and pulmonary sequelae in COVID-19.

## Methods

### Participants

The internal review board approved this retrospective study. Written informed consent was waived with approval. Between January 22, and March 16, 2020, 172 laboratory-confirmed COVID-19 patients who underwent chest CT were collected from eight hospitals in China. The cases were from four regions (Xi'an, *n* = 80; Baoji, *n* = 10; Ankang, *n* = 18; Hanzhong, *n* = 17) in Shaanxi province and Wuhan (*n* = 47) in Hubei province.

A case of COVID-19 was confirmed by a positive result on next-generation sequencing or real-time RT-PCR. The disease type, i.e., uncomplicated illness, mild pneumonia, severe pneumonia, critical illness (acute respiratory distress syndrome, sepsis or septic shock) was evaluated based on the criteria published by World Health Organization (WHO) (20).

All the patients were treated based on Diagnosis and Treatment Protocol for Novel Coronavirus Pneumonia (Trial Version 7) issued by National Health Commission of the People's Republic of China, which includes initiation of antivirals, interferon, Chinese herbal medications, supplemental oxygen as needed and hospitalization. The criteria for patient discharge with recovery included: (1) afebrile for >3 days, (2) improved respiratory symptoms, (3) chest imaging shows obvious resolution of inflammation, and (4) two consecutively negative nucleic acid test results (sampling interval ≥1 day) (21). The recommendations for discharged patients included (1) 14 days of isolation management and health monitoring; (2) follow-up hospital visits with a next-generation sequencing or real-time RT-PCR test and chest CT scan to detect whether there exist a positive return and/or pulmonary residuals excluding the underlying lesions on CT with linear opacities, and/or a few consolidation with/without GGO at 2 and 4 weeks after discharge (21).

### CT Image Acquisition

All chest CT were acquired by using 16- or 64-multidector CT scanners (GE LightSpeed 16, GE VCT LightSpeed 64, GE Optima 680, GE Healthcare; Philips Brilliant 16, Philips Healthcare; Somatom Sensation 64, Somatom AS, Somatom Spirit, Siemens Healthcare). Patients were scanned in the supine position from the level of the upper thoracic inlet to the inferior level of the costophrenic angle with the following parameters: tube voltage of 120 kVp, current intelligent control (auto mA) of 30–300 mA, and slice thickness reconstructions of 0.625–1.5 mm.

### Data Collection and Evaluation

We extracted the demographic data, clinical symptoms, and laboratory tests on admission from electronic medical records. The date of disease onset was defined as patients' reported date of symptom onset. The time intervals from symptom onset to each CT were determined. The primary clinical outcome was discharge or adverse outcome (admission to ICU, use of mechanical ventilation, or death). The secondary outcome was pulmonary sequelae, i.e., complete absorption or residuals on CT at the first follow-up visit after discharge.

All CT images and pattern categorization were independently evaluated by two experienced radiologists, respectively, with 4 and 10 years of pulmonary imaging experience, who were blinded to the clinical and laboratory data of patients. Prior to the evaluation, they were trained by a lecture- and literature-based session that explained CT findings (10–13), a chest imaging score assessing the degree of lobar involvement (22), and pattern categorizations (15, 17) of COVID-19. During the session, 209 CT images from 56 cases randomly selected from this study cohort were individually evaluated and then differences were discussed with a final consensus. The remaining CT images were first individually evaluated and then evaluated together 3 weeks after individual evaluation. Any difference was discussed with a final consensus. Individual evaluations were used for calculation of inter-observer agreement (see more in the Supplementary Material), and consensus evaluations were used for subsequent analysis.

CT findings including the presence and distribution of GGO, consolidation, linear opacity, pleural effusion and lymphadenopathy were evaluated. The degree of lobar involvement and total lung severity score were also evaluated (22). Based on the degree or area of involvement, each of the five lung lobes was scored of 0 for 0% lobe involvement, 1 for 1–25% lobe involvement, 2 for 26–50% lobe involvement, 3 for 51–75% lobe involvement, or 4 for 76–100% lobe involvement. A total severity score was calculated by summing the scores of the five lobes (range, 0–20).

CT pattern categorization was performed based on the above CT findings and total lung severity (15, 17) (Table 1). Receiver operating characteristic curve analysis was used to estimate the cutoff CT scores in discriminations of Pattern 2 vs. 3 and Pattern 3 vs. 4, respectively (see more in Supplement Material). In cases with two or more patterns, predominant pattern was designated.

**Table 1:** Definition of COVID-19 pneumonic pattern based on CT findings.

**CT pattern**	**Definition**	**CT findings**
Pattern 0	Negative	None
Pattern 1	Bronchopneumonia pattern	• Discrete lesion with a peribronchial distribution • CT signs with GGO or consolidation, or tree-in-bud sign or nodular opacity (Figure 3) • Lung lobar involvement assessed by total CT score ≤ 5
Pattern 2	Organizing pneumonia pattern	• Multifocal lesions with a peripheral distribution predominantly in the middle to lower lung zones • CT signs with GGO or consolidation, and/or interlobular septal thickening (Figure 4) • Lung lobar involvement assessed by total CT score ≤ 6
Pattern 3	Progressive organizing pneumonia pattern	• Multiple lesions with a peripheral distribution predominantly in the middle to lower lung zones • CT signs with consolidation or GGO or mixed GGO and consolidation, and/or interlobular septal thickening (Figure 5) • Lung lobar involvement assessed by total CT score more than 6 and <10
Pattern 4	Diffuse alveolar damage pattern	• Lesions with extensive distribution diffusely in the entire lungs • CT signs with consolidation mixed with or without GGO, and/or air bronchograms (Figure 6) • Lung lobar involvement assessed by total CT score more than or equal to 10

*The primary CT signs (GGO, consolidation, linear opacity, interlobular septal thickening and air bronchograms) were included to define the CT patterns; while other signs e.g.„ pleural effusion, lymphadenopathy and so on were not considered due to the infrequency in each pattern. Negative refers to the no abnormality on CT*.

*GGO, ground glass opacities*.

### Statistical Analysis

Continuous variables were represented as means and standard deviations, while categorical variables were expressed as counts and percentages. Differences of demographic, clinical and CT imaging characteristics across pattern groups were analyzed by dependent sample *t*-test, Chi-square test or Fisher's exact test as appropriate. Bonferroni correction was used in multiple comparisons. Chi-square test for trend was used to explore the time-dependent change of each CT pattern. Univariate Cox proportional-hazards regression was first used to explore the risk factors related to clinical adverse outcomes and pulmonary residuals. Multivariate Cox proportional-hazards regression with Kaplan-Meier curve plots were further used to explore the risk factors based on the significant variables in the above univariate analysis.

All statistical analyses were performed using SPSS 17.0 (SPSS; Chicago, IL, USA) and Medcalc 19.1.7 (MedCals Software Ltd.; Ostend, Belgium). *P* < 0.05 was considered statistically significant.

## Result

### Patient Demographic and Clinical Characteristics

**Figure 1:**
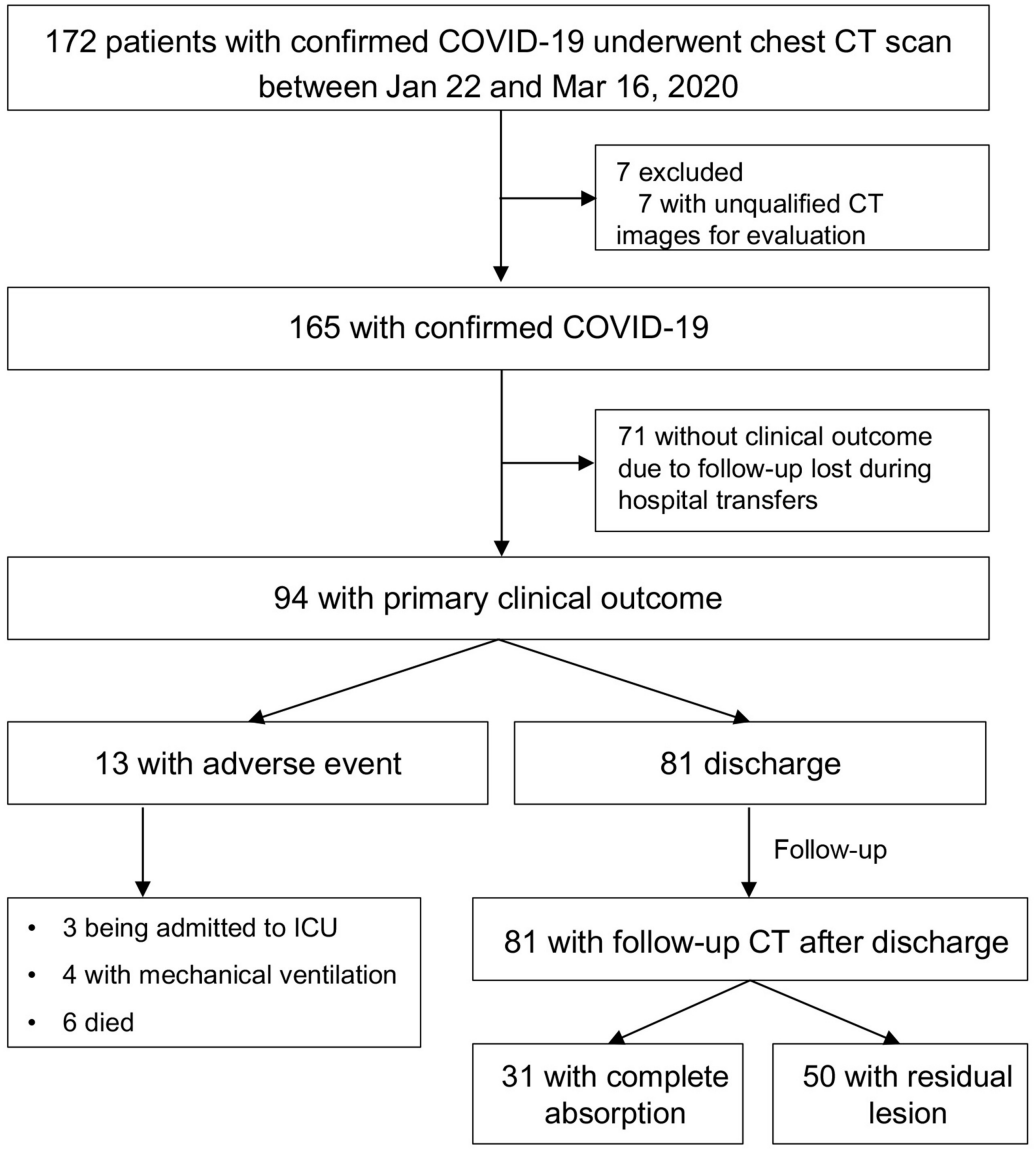
Study flow diagram. COVID-19, coronavirus disease 2019; ICU, intensive care unit.

Of 172 patients, 165 patients were included. As of 16 Mar 2020, 94 patients had clinical outcomes and 71 were follow-up lost without clinical outcome records due to hospital transfer (Figure 1). Of 94 patients, 81(86.2%) were discharged, 3(3.2%) were admitted to ICU, 4(4.3%) required mechanical ventilation, 6(6.4%) died. 31(38.3%) patients had complete absorption of lesions on CT after discharge. The median time from symptom onset to discharge was 21 (range, 10–41) days, and median times from symptom onset to being admitted to ICU, to requiring mechanical ventilation, and to death were 7 (range, 2–8) days, 8 (range, 8–49) days, and 33.5 (range, 7–39) days, respectively. The median times from symptom onset and from discharge to post-discharge CT scan were 37 (range, 14–58) days, 15 (range, 9–29) days, respectively.

Patients were categorized into five CT patterns based on the baseline CT: 7(4.3%) were Pattern 0, 36 (21.8%) were Pattern 1, 67 (40.6%) were Pattern 2, 32 (19.4%) were Pattern 3, and 23(13.9%) were Pattern 4. All the patients had 478 chest CT, 34 (21.2%) had 1 CT, 41 (23.6 %) had 2 CT, 39 (23.7%) had 3 CT, and 51 (31.5%) had more than 3 CT. The median time from symptom onset to baseline CT was 7 (range, 1–44) days.

Table 2 detailed the clinical characteristics and laboratory results of patients by CT pattern group. In the full cohort, the mean age was 49.5 (SD, 15.9; range, 4–89) years and there was no gender difference [91 [55.2%] men, 74 [44.8%] women]. Significant differences between pattern groups were found in age, sex distribution, disease severity, comorbidity, CT findings and laboratory results (all *P* < 0.05). Significant differences were also observed in multiple comparisons between any two patterns in one or more than one terms of age, sex distribution, disease severity, comorbidity, CT findings and laboratory results (all *P* < 0.017).

**Table 2:** Characteristics of COVID-19 pneumonia patients with various CT patterns.

**Characteristic**	**All (*n* = 165)**	**Pattern 0 (*n* = 7)**	**Pattern 1 (*n* = 36)**	**Pattern 2 (*n* = 67)**	**Pattern 3 (*n* = 32)**	**Pattern 4 (*n* = 23)**	***P*-value**	**Pattern 0 vs. Pattern 1**	**Pattern 1 vs. Pattern 2**	**Pattern 2 vs. Pattern 3**	**Pattern 3 vs. Pattern 4**
								***P*-value**	***P*-value**	***P*-value**	***P*-value**
Age (years) ^a^	49.5 ± 15.9	39.7 ± 13.7	47.4 ± 16.5	43.9 ± 14.7	56.7 ± 11.1	61.7 ± 14.7	**<** **0.001**	0.253	0.266	**<** **0.001**^**†**^	0.158
Male sex	91 (55.2)	3 (42.9)	26 (72.2)	28 (41.8)	17 (53.1)	17 (73.9)	**0.012**	0.129	**0.003**^**†**^	0.289	0.118
Disease severity							**<** **0.001**	0.294	0.949	**<** **0.001**^**†**^	0.014
Mild	111 (67.3)	7 (100)	31 (86.1)	58 (86.6)	13 (40.6)	2 (8.7)					
Severe	44 (26.7)	0	5 (13.9)	9 (13.4)	16 (50.0)	14 (60.9)					
Critical illness	10 (6.0)	0	0	0	3 (9.4)	7 (30.4)					
Comorbidity ^b^	101 (61.2)	2 (28.6)	8 (22.2)	21 (31.3)	18 (56.2)	15 (65.2)	**0.002**	0.716	0.326	0.018	0.503
Clinical symptom on admission											
Fever	140 (84.8)	4 (57.1)	26 (72.2)	60 (89.6)	28 (87.5)	22 (95.7)	**0.020**	0.655	0.024	0.743	0.387
Fatigue	30 (18.2)	3 (42.9)	2 (5.6)	11 (16.4)	5 (15.6)	9 (39.1)	**0.008**	0.024	0.133	0.920	0.048
Pharyngalgia	18 (10.9)	2 (28.6)	4 (11.1)	9 (13.4)	2 (6.3)	1 (4.3)	0.347	0.248	>0.999	0.495	>0.999
Headache	6 (3.6)	0	2 (5.6)	4 (6.0)	0	0	0.602	>0.999	>0.999	0.301	–
Cough	96 (58.2)	4 (57.1)	16 (44.4)	42 (62.7)	17 (53.1)	17 (73.9)	0.195	0.687	0.075	0.365	0.118
Expectoration	36 (21.8)	1 (14.3)	6 (16.7)	18 (26.9)	3 (9.4)	8 (34.8)	0.129	>0.999	0.243	0.046	0.038
Chest congestion/breath shortness	34 (20.6)	0	2 (5.6)	9 (13.4)	13 (40.6)	10 (43.5)	**<** **0.001**	>0.999	0.321	**0.002**^**†**^	0.832
Muscle soreness	8 (4.8)	0	2 (5.6)	4 (6.0)	1 (3.1)	1 (4.3)	>0.999	>0.999	>0.999	>0.999	>0.999
Nausea and vomiting	1 (0.6)	0	1 (2.8)	0	0	0	>0.999	–	>0.999	>0.999	–
Diarrhea	4 (2.4)	0	0	2 (3.0)	1 (3.1)	1 (4.3)	0.735	–	0.541	>0.999	>0.999
No symptom	5 (3.0)	0	3 (8.3)	2 (3.0)	0	0	0.365	>0.999	0.340	>0.999	–
Laboratory test on admission ^c^											
Lymphocyte percentage (%)							**<** **0.001**	0.280	0.097	**0.004**^**†**^	0.836
<20	62 (38.0)	0	6 (16.7)	21 (31.8)	20 (62.5)	15 (65.2)					
≥20	101 (62.0)	6 (100)	30 (83.3)	45 (68.2)	12 (37.5)	8 (34.8)					
Monocyte percentage (%)							0.315	0.414	0.085	0.102	0.261
>10	39 (24.5)	1 (16.7)	12 (33.3)	12 (18.2)	10 (33.3)	4 (19.0)					
≤ 10	120 (75.5)	5 (83.3)	24 (66.7)	54 (81.8)	20 (66.7)	17 (81.0)					
Leukocyte count (10^9^/L)							0.062	0.167	0.570	0.924	0.014
<3.5	40 (24.5)	0	9 (25.0)	20 (30.3)	10 (31.2)	1 (4.3)					
≥3.5	123 (75.5)	6 (100)	27 (75.0)	46 (69.7)	22 (68.8)	22 (95.7)					
Alanine Aminotransferase (U/L)							0.065	0.554	0.102	0.200	0.945
>50	28 (17.4)	0	2 (5.6)	11 (16.9)	9 (28.1)	6 (27.3)					
≤ 50	133 (82.6)	6 (100)	34 (94.4)	54 (83.1)	23 (71.9)	16 (72.7)					
Aspartate Aminotransferase (U/L)							0.122	0.328	0.035	0.702	0.583
>40	32 (19.9)	1 (16.7)	2 (5.6)	14 (21.5)	8 (25.0)	7 (31.8)					
≤ 40	129 (80.1)	5 (83.3)	34 (94.4)	51 (78.5)	24 (75.0)	15 (68.2)					
Creatine kinase (U/L)							**0.014**	0.014	0.022	0.429	0.038
>310	18 (11.8)	1 (16.7)	0	9 (13.6)	2 (7.7)	6 (31.5)					
≤ 310	134 (88.2)	5 (83.3)	35 (100)	57 (86.4)	24 (92.3)	13 (68.4)					
Neutrophil percentage (%)							**<** **0.001**	0.391	0.080	0.232	0.043
>75	48 (29.4)	0	4 (11.1)	17 (25.8)	12 (37.5)	15 (65.2)					
≤ 75	115 (70.6)	6 (100)	32 (88.9)	49 (74.2)	20 (62.5)	8 (34.8)					
C-reactive protein (mg/L)							**0.002**	0.130	0.245	0.356	0.055
>10	96 (63.6)	1 (16.7)	17 (50.0)	38 (62.3)	23 (71.9)	17 (94.4)					
≤ 10	55 (36.4)	5 (83.3)	17 (50.0)	23 (37.7)	9 (28.1)	1 (5.6)					
Hemoglobin (g/L)							0.494	0.873	0.976	0.684	0.251
<130	35 (22.4)	1 (16.7)	7 (19.4)	13 (19.7)	7 (23.3)	7 (38.9)					
≥130	121 (77.6)	5 (83.3)	29 (80.6)	53 (80.3)	23 (76.7)	11 (61.1)					
CT findings on admission											
CT signs											
GGO only	28 (17.0)	0	13 (36.1)	12 (17.9)	2 (6.3)	1 (4.3)	**0.005**	**–**	0.040	0.215	>0.999
Consolidation	17 (10.3)	0	5 (13.9)	6 (9.0)	3 (9.4)	3 (13.0)	0.880	**–**	0.510	>0.999	0.686
GGO and consolidation	51 (30.9)	0	10 (27.8)	16 (23.9)	10 (31.3)	15 (65.2)	**0.002**	**–**	0.664	0.436	**0.013**
Linear opacity	0	0	0	0	0	0	–	**–**	–	–	**–**
GGO and linear opacity	7 (4.2)	0	2 (5.6)	3 (4.5)	2 (6.3)	0	0.839	**–**	>0.999	0.657	0.504
Consolidation and linear opacity	5 (3.0)	0	1 (2.8)	4 (6.0)	0	0	0.618	**–**	0.665	0.301	–
Three mixed signs	50 (30.3)	0	5 (13.9)	26 (38.8)	15 (46.9)	4 (17.4)	**0.003**	**–**	**0.009**^**†**^	0.446	0.023
Lobe involvement							**<** **0.001**	**–**	0.121	**0.008**^**†**^	0.632
Number of lobe affected <3	52 (31.5)	7 (100)	18 (50.0)	23 (34.3)	3 (9.4)	1 (4.3)					
Number of lobe affected≥3	113 (68.5)	0	18 (50.0)	44 (65.7)	29 (90.6)	22 (95.7)					
CT severity score ^a^	6.0 ± 4.4	0	3.3 ± 2.1	4.7 ± 2.7	7.5 ± 2.8	14.0 ± 2.9	**<** **0.001**	**<** **0.001**^**†**^	**0.005**^**†**^	**<** **0.001**^**†**^	**<** **0.001**^**†**^

*Unless otherwise indicated, data are reported as the number of patients, with percentages in parentheses. a, data are reported as the mean ± standard derivation. b, 70% of patients had history of hypertension and diabetes mellitus while only 2 had pulmonary tuberculosis and 2 had chronic bronchitis. c, more than 91·5% of patients had all laboratory tests and a few were lack of one or two indicators*.

†, *Significance at P < 0.0125 with Bonferroni correction. Abbreviations: Pattern 0 = negative; Pattern 1 = organizing pneumonia pattern; Pattern 2 = progressive organizing pneumonia pattern; Pattern 4 = diffuse alveolar damage pattern; GGO, ground glass opacity; Three mixed signs = GGO, consolidation and linear opacity. The bold value refers to P < 0.05*.

### Evolution of COVID-19 Pneumonic CT Pattern With Disease Progression

**Figure 2:**
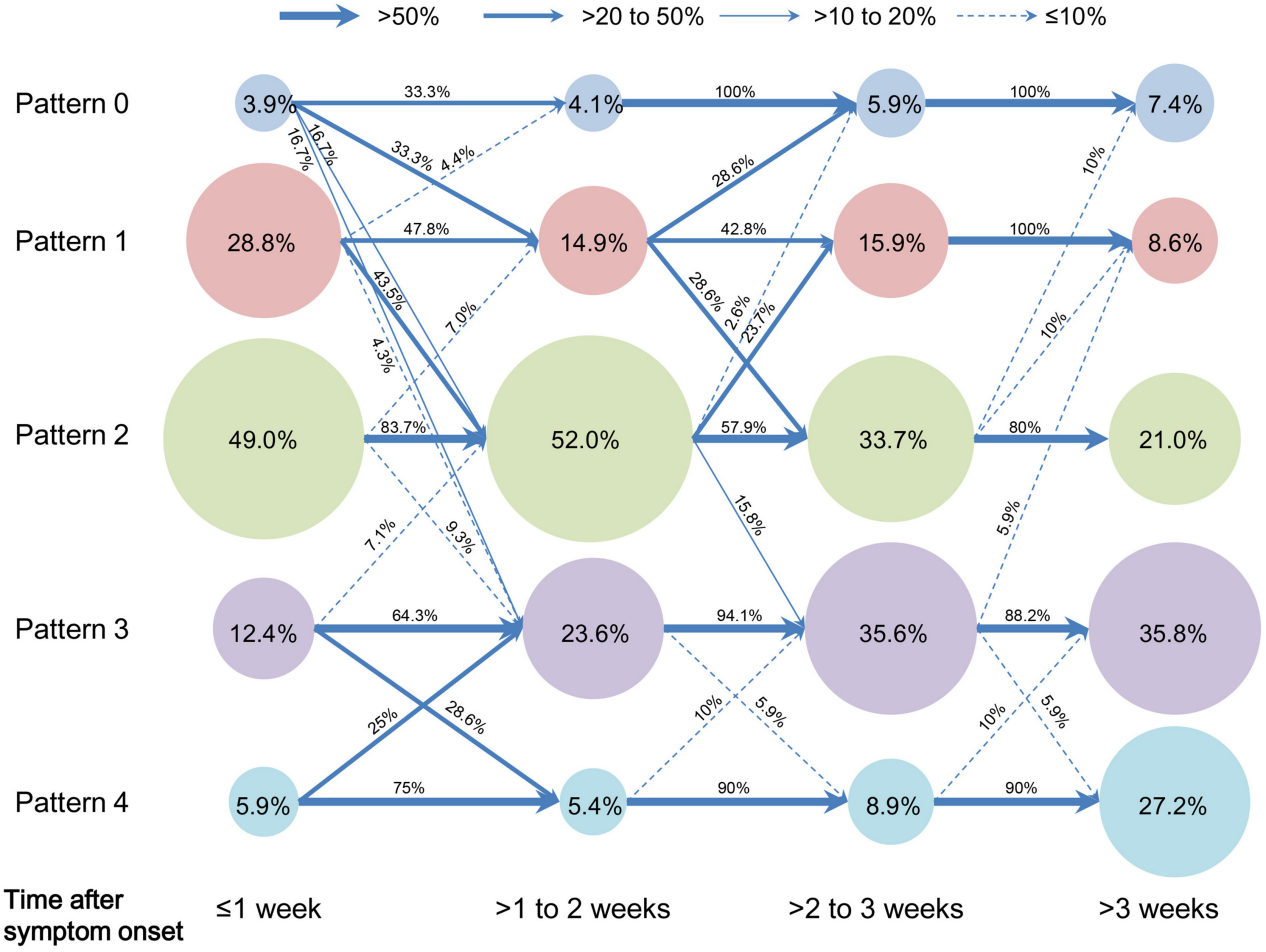
Evolution of proportions of COVID-19 pneumonic CT pattern with the disease progression. Pattern data were designated to four time groups according to the time from symptom onset to CT scan: ≤ 1 week (CT pattern number = 153), >1–2 weeks (CT pattern number = 147), >2–3 weeks (CT pattern number = 101) and >3 weeks (CT pattern number = 77). The circular area indicated the proportion of CT pattern in each time group, e.g.„ proportions of Pattern 0 to 4 were 3.9, 28.8, 49.0, 12.4, and 5.9% during 1 week after symptom onset, respectively. Arrow line indicated the evolution of each CT pattern from a time group to the following, e.g.„ 33.3% of Pattern 1 progressed to Pattern 2 from 1 to 2 weeks after symptom onset; here four arrow line style denoted the categorization of evolution proportion, i.e.„ >50% (thick solid line), >20–50% (medium solid line), >10–20% (thin solid line), and ≤ 10% (dashed line).

Chi-square tests for trend indicated that as disease progresses from 1 to >3 weeks, proportions of Pattern 1 and 2 remarkably decreased, while those of Pattern 3 and 4 increased (all *P* < 0.01). With regard to evolution of CT pattern, Pattern 0–2 showed a remarkable evolution with overlaps of progression and downgrade within 3 weeks after symptom onset, and mostly remained the same thereafter. Pattern 3 and 4 showed a remarkable evolution (progression or downgrade) within 2 weeks, and most of them remained afterwards (Figure 2).

**Figure 3:**
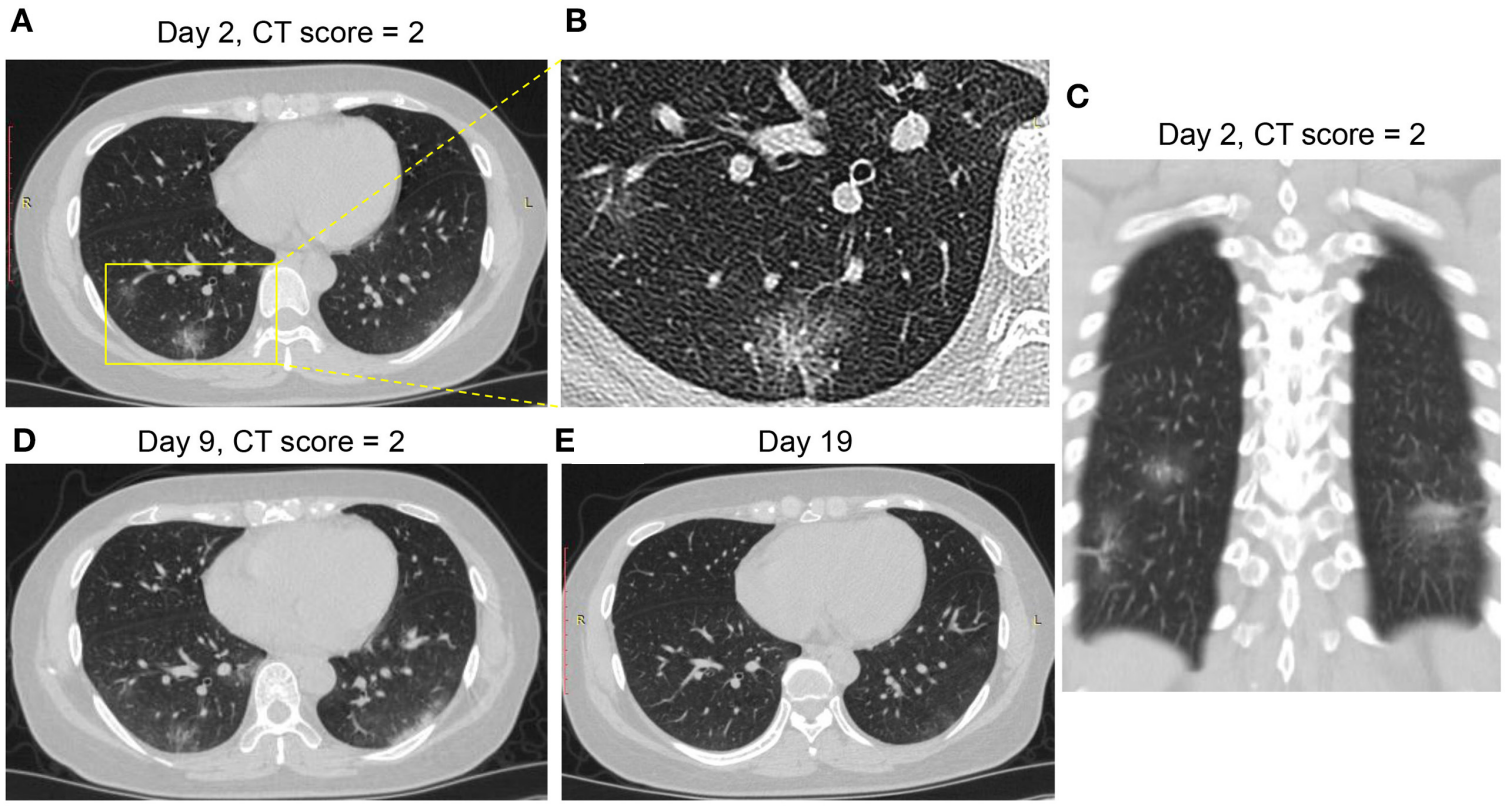
CT Pattern 1 (bronchopneumonia pattern) in a 38-year-old woman with COVID-19 pneumonia who was admitted to hospital at day 2 after symptom onset. **(A–C)** Axial and coronal CT images demonstrate multifocal peribronchial ground-glass opacity (GGO) at day 2; Axial CT images demonstrate increasing density and size of lesions at day 9 **(D)** and subsequently complete absorption at day 19 **(E)**.

**Figure 4:**
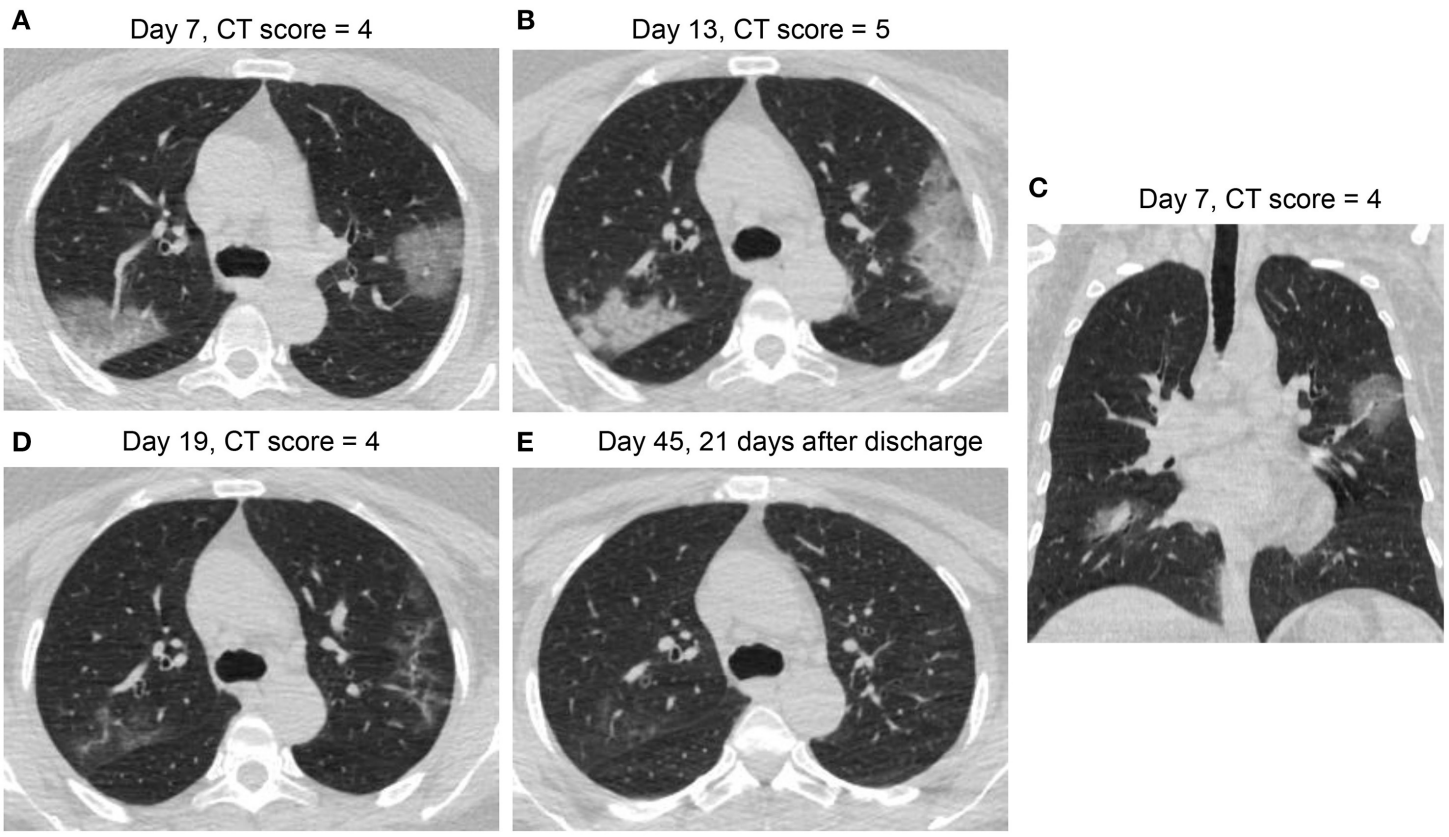
CT Pattern 2 (organizing pneumonia pattern) in a 49-year-old woman with COVID-19 pneumonia who was admitted to hospital at day 7 after symptom onset and discharged at day 24. **(A,C)** Axial and coronal CT images demonstrate multifocal ground-glass opacity (GGO), mixed GGO and consolidation at day 7; Axial CT images demonstrate consolidation at day 13 **(B)**, subsequent absorption with mixed GGO and linear opacities at day 19 **(D)**, and complete absorption at day 45 **(E)**.

**Figure 5:**
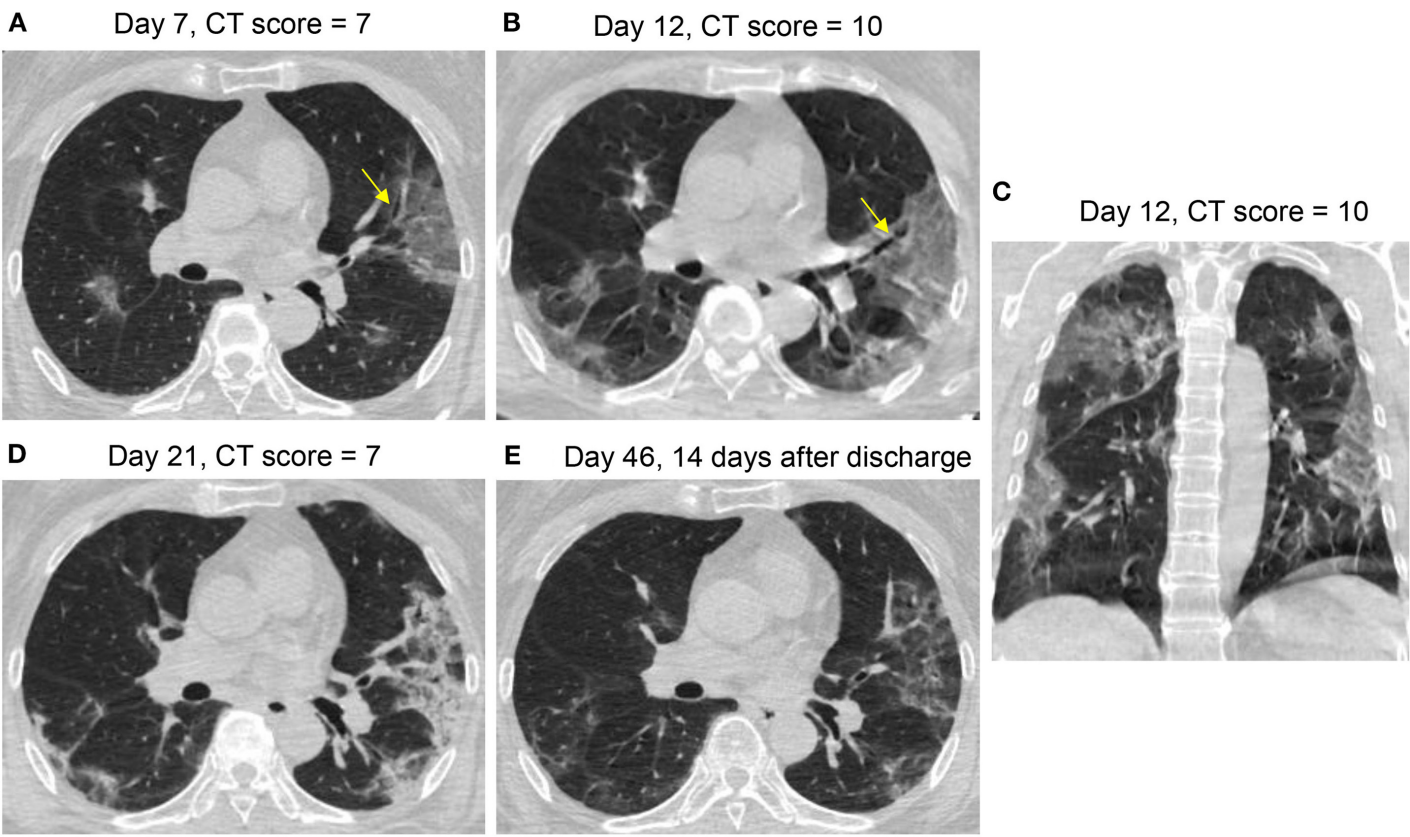
CT Pattern 3 (progressive organizing pneumonia pattern) in a 65-year-old woman with COVID-19 pneumonia who was admitted to hospital at day 7 after symptom onset and discharged at day 24. Axial and coronal CT images demonstrate a fast progression from patchy ground-glass opacity (GGO) with slight bronchial dilatation (arrow) at day 7 **(A)**, to extensive GGO and consolidation with progressive bronchial dilatation (arrow) at day 12 **(B,C)**; Axial CT images reveal that extensive GGO and consolidation turned into consolidation and reticulation at day 21 **(D)** and into mixed GGO and linear opacities at day 46 **(E)**.

**Figure 6:**
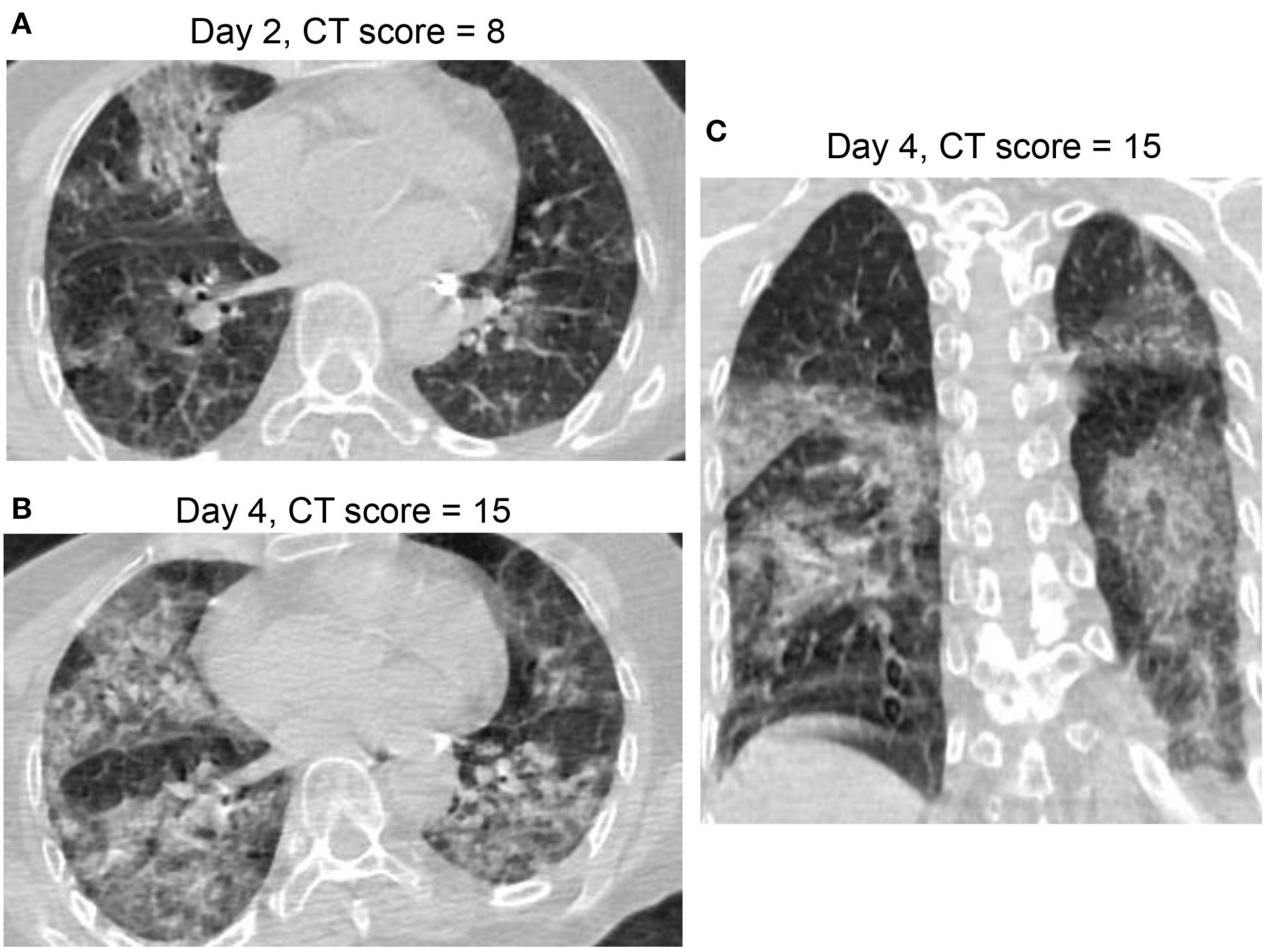
CT Pattern 4 (diffuse alveolar damage pattern) in an 82-year-old woman COVID-19 pneumonia and with history of cardiovascular disease and chronic obstructive pulmonary disease, who was admitted to intensive care unit with mechanical ventilation at day 7 after symptom onset and died at day 39. Axial CT images demonstrate a fast progression from mixed ground-glass opacity (GGO) and consolidation at day 2 **(A)** to a geographic distribution of mixed consolidation and interlobular septal thickening at day 4 **(B)**; **(C)** Coronal CT image demonstrates mixed consolidation and interlobular septal thickening with diffused distribution of both lungs.

Figures 3–6 presented CT findings with disease progression in Pattern 1 to 4 cases. Pattern 1 and 2 showed limited progression with increasing density and size of lesions from 1 to 2 weeks after onset, while had complete absorption subsequently. Pattern 3 showed a fast progression from patchy GGO to extensively mixed GGO and consolidation within 2 weeks, and subsequently turned into mixed GGO and linear opacities. Pattern 4 showed a considerably fast progression to diffusely mixed consolidation and interlobular septal thickening in both lungs and had adverse outcome within 1 week.

### Prognostic Significance of Pneumonic CT Pattern in COVID-19

Supplementary Table 1 detailed the clinical, laboratory and CT imaging characteristics of patients in clinical outcome and pulmonary sequelae on CT. Significant differences between discharge and adverse outcome were found in age, disease severity, comorbidity, laboratory results, CT pattern and CT score (all *P* < 0.05). For pulmonary sequelae, significant differences between complete absorption and residuals were found in age, elevated neutrophil percentage, elevated C-reactive protein, CT pattern and CT score (all *P* < 0.05).

### Correlations of CT Pattern With Clinical Outcomes

**Figure 7:**
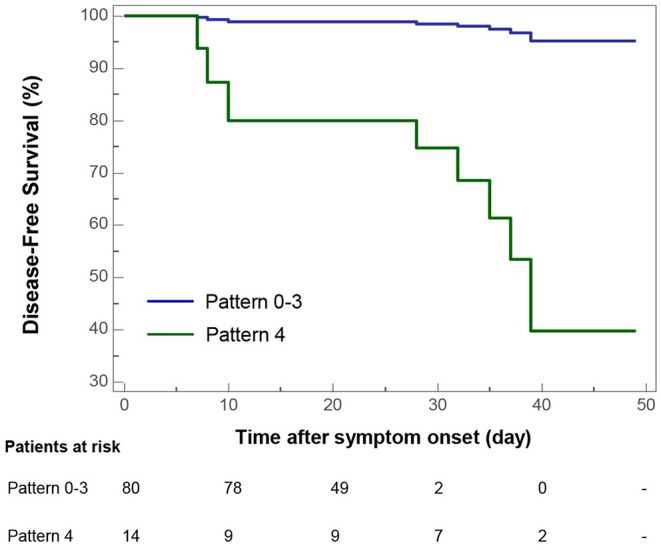
Kaplan-Meier curve plots showing time from symptom onset to adverse outcome events (admission to intensive care unit, use of mechanical ventilation, or death) by categories of COVID-19 pneumonic CT pattern (Pattern 4 vs. Pattern 0–3 as reference).

Univariate Cox proportional-hazards regression indicated that CT Pattern 4 [Hazard ratio [HR] 36.67, 95% confidence interval [95% CI] 4.38–307.25, *P* = 0.001] significantly correlated with adverse outcomes. Besides, age ≥65 years (HR 9.39, 95% CI 2.38–37.11, *P* = 0.001), comorbidity (HR 4.14, 95% CI 1.09–15.71, *P* = 0.037), severe or critical illness (HR 4.62, 95% CI 2.04–10.46, *P* < 0.001), presence of fatigue (HR 3.62, 95% CI 1.16–11.28, *P* = 0.027) and chest congestion and/or shortness of breath (HR 3.81, 95% CI 1.19–12.18, *P* = 0.024), neutrophil percentage >75% (HR 14.12, 95% CI 1.75–114.21, *P* = 0.013), CT score ≥10 (HR 11.66, 95% CI 2.31–58.75, *P* = 0.003) were associated with adverse outcomes (Table 3). Multivariate analysis indicated that after controlling for age, Pattern 4 was found to be an independent risk factor for adverse outcomes (HR 18.90, 95% CI 1.91–186.60, *P* = 0.012) (Figure 7).

**Table 3:** Risk factors associated with adverse outcome in patients with COVID-19 pneumonia.

**Variable**	**Stratification**	**Univariate analysis**			**Multivariate analysis**		
		**HR**	**95% CI**	***P*-value**	**HR**	**95% CI**	***P*-value**
Age (years)	≥65 vs. <65 (Ref.)	9.39	2.38–37.11	**0.001**	3.04	0.74–12.56	0.124
Sex	Male vs. female (Ref.)	0.86	0.27–2.77	0.805			
Comorbidity	Yes vs. No (Ref.)	4.14	1.09–15.71	**0.037**			
Disease severity	Severe, critical illness vs. Mild (Ref.)	4.62	2.04–10.46	**<** **0.001**			
Laboratory test at admission							
Lymphocyte percentage (%)	<20 vs. ≥20 (Ref.)	1.00	0.24–4.16	0.998			
Monocyte percentage (%)	>10 vs. ≤ 10 (Ref.)	0.33	0.04–2.60	0.294			
Leukocyte count (10^9^/L)	<3.5 vs. ≥3.5 (Ref.)	0.03	0–76.60	0.390			
Alanine Aminotransferase (U/L)	>50 vs. ≤ 50 (Ref.)	0.82	0.21–3.16	0.820			
Aspartate Aminotransferase (U/L)	>40 vs. ≤ 40 (Ref.)	2.01	0.63–6.40	0.239			
Creatine kinase (U/L)	>310 vs. ≤ 310 (Ref.)	3.39	0.87–13.18	0.078			
Neutrophil percentage (%)	>75 vs. ≤ 75 (Ref.)	14.12	1.75–114.21	**0.013**			
C-reactive protein (mg/L)	>10 vs. ≤ 10 (Ref.)	53.87	0.12–2.5 × 10^4^	0.203			
Hemoglobin (g/L)	<130 vs. ≥130 (Ref.)	0.69	0.17–2.83	0.606			
CT findings							
GGO only	Yes vs. No (Ref.)	2.79	0.34–23.19	0.343			
Consolidation	Yes vs. No (Ref.)	0.04	0–6781	0.607			
GGO and consolidation	Yes vs. No (Ref.)	3.24	0.93–11.27	0.065			
Linear opacity	Yes vs. No (Ref.)	— -	— -	— -			
GGO and linear opacity	Yes vs. No (Ref.)	0.04	0–2.3 × 10^4^	0.641			
Consolidation and linear opacity	Yes vs. No (Ref.)	0.05	0–1.7 × 10^6^	0.730			
Three mixed signs	Yes vs. No (Ref.)	0.47	0.13–1.74	0.255			
Number of lobe affected	>3 vs. ≤ 3 (Ref.)	4.86	0.59–39.77	0.141			
CT severity score	≥10 vs. <10 (Ref.)	11.66	2.31–58.75	**0.003**			
CT pattern	Pattern 4 vs. Pattern 0–3 (Ref.)	36.67	4.38–307.25	**0.001**	18.90	1.91–186.60	**0.012**

*Ref. refers to the stratification of variable as reference in the Cox hazard-proportional regression analysis*.

*HR, hazard ratio; 95% CI, 95% confidence interval; GGO, ground glass opacity; Three mixed signs, GGO, consolidation and linear opacity; Pattern 0, negative; Pattern 1, organizing pneumonia pattern; Pattern 2, progressive organizing pneumonia pattern; Pattern 4, diffuse alveolar damage pattern. The bold value refers to P < 0.05*.

### Correlations of CT Pattern With Pulmonary Sequelae on CT After Discharge

**Figure 8:**
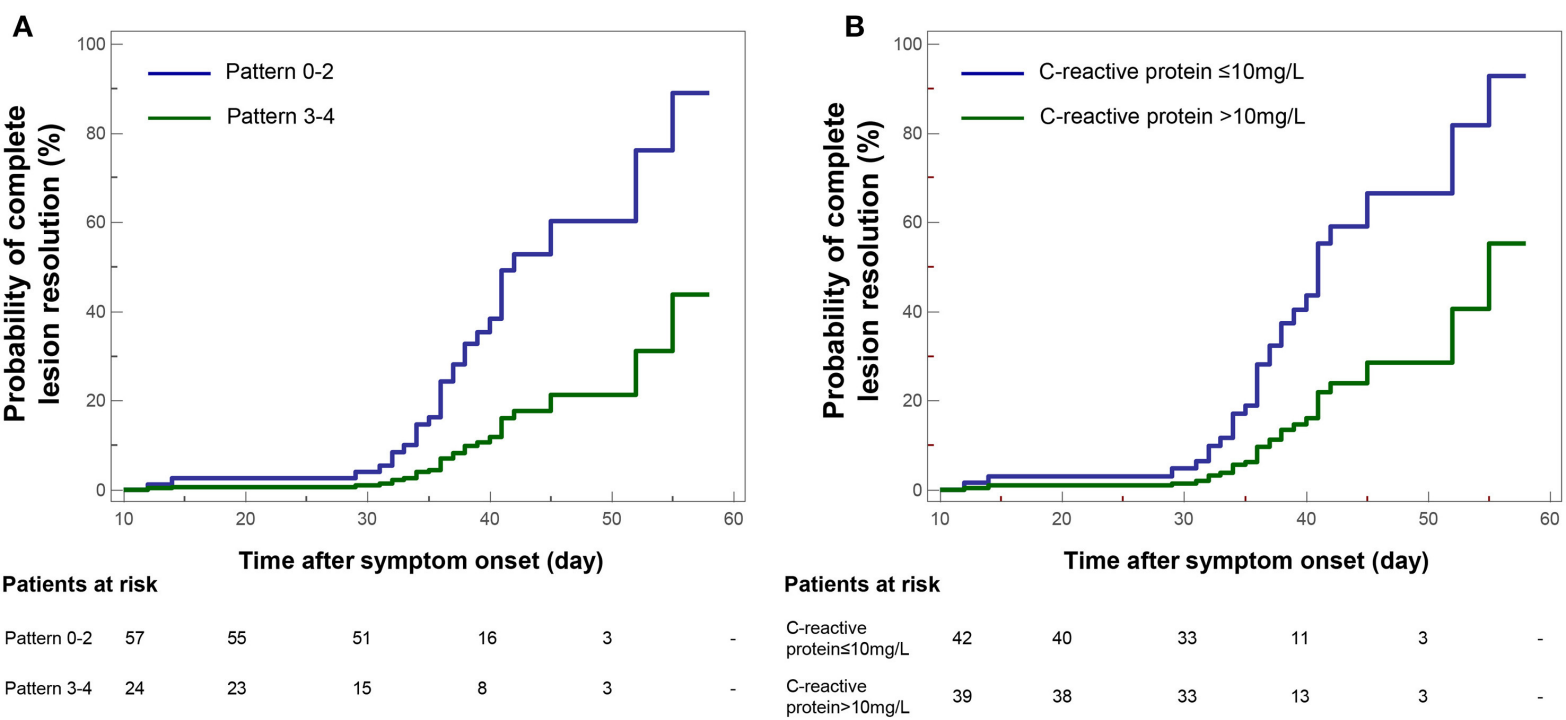
Kaplan-Meier curve plots showing time from symptom onset to complete resolution of pulmonary lesions by **(A)** categories of COVID-19 pneumonic CT pattern (Pattern 3–4 vs. Pattern 0–2 as reference), and **(B)** conditions of C-reactive protein.

By univariate Cox proportional-hazards regression, it was found that CT Pattern 3 or 4 (HR 0.23, 95% CI 0.07–0.78, *P* = 0.017) were significantly related with pulmonary sequelae. Beyond, significant factors included age ≥45 years (HR 0.36, 95% CI 0.15–0.88, *P* = 0.025), C-reactive protein concentration >10 mg/L (HR 0.28, 95% CI 0.12–0.65, *P* = 0.003), number of lobe affected >3 (HR 0.34, 95% CI 0.16–0.71, *P* = 0.005), CT score ≥4 (HR 0.32, 95% CI 0.15–0.65, *P* = 0.002) (Table 4). The multivariate analysis showed that Pattern 3 or 4 (HR 0.26, 95% CI 0.08–0.88, *P* = 0.030) and C-reactive protein (HR 0.31, 95% CI 0.13–0.72, *P* = 0.006) were two independent factors associated with pulmonary residuals (Figure 8).

**Table 4:** Risk factors associated with pulmonary sequelae of lesion resolution at 2–3 weeks after discharge in patients with COVID-19 pneumonia.

**Variable**	**Stratification**	**Univariate analysis**			**Multivariate analysis**		
		**HR**	**95% CI**	***P*-value**	**HR**	**95% CI**	***P*-value**
Age (yr)	≥45 vs. <45 (Ref.)	0.36	0.15–0.88	**0.025**			
Sex	Male vs. Female (Ref.)	1.09	0.53–2.25	0.806			
Comorbidity	Yes vs. No (Ref.)	0.46	0.18–1.21	0.116			
Disease severity	Severe vs. Mild (Ref.)	0.87	0.12–6.43	0.893			
Laboratory test at admission							
Lymphocyte percentage (%)	<20 vs. ≥20 (Ref.)	0.50	0.22–1.13	0.094			
Monocyte percentage (%)	>10 vs. ≤ 10 (Ref.)	1.94	0.92–4.09	0.082			
Leukocyte count (10^9^/L)	<3.5 vs. ≥3.5 (Ref.)	0.96	0.39–2.38	0.928			
Alanine Aminotransferase (U/L)	>50 vs. ≤ 50 (Ref.)	0.50	0.17–1.46	0.202			
Aspartate Aminotransferase (U/L)	>40 vs. ≤ 40 (Ref.)	0.69	0.27–1.81	0.451			
Creatine kinase (U/L)	>310 vs. ≤ 310 (Ref.)	0.50	0.12–2.12	0.349			
Neutrophil percentage (%)	>75 vs. ≤ 75 (Ref.)	0.32	0.10–1.06	0.062			
C-reactive protein (mg/L)	>10 vs. ≤ 10 (Ref.)	0.28	0.12–0.65	**0.003**	0.31	0.13–0.72	**0.006**
Hemoglobin (g/L)	<130 vs. ≥130 (Ref.)	0.36	0.09–1.54	0.169			
CT findings							
GGO only	Yes vs. No (Ref.)	1.14	0.34–3.84	0.827			
Consolidation	Yes vs. No (Ref.)	2.89	1.08–7.72	**0.035**			
GGO and consolidation	Yes vs. No (Ref.)	1.02	0.45–2.28	0.969			
Linear opacity	Yes vs. No (Ref.)	–	–	–			
GGO and linear opacity	Yes vs. No (Ref.)	0.88	0.21–3.71	0.856			
Consolidation and linear opacity	Yes vs. No (Ref.)	0.89	0.12–6.59	0.911			
Three mixed signs	Yes vs. No (Ref.)	0.52	0.24–1.13	0.098			
Number of lobe affected	>3 vs. ≤ 3 (Ref.)	0.34	0.16–0.71	**0.005**			
CT severity score	≥4 vs. <4 (Ref.)	0.32	0.15–0.65	**0.002**			
CT Pattern	Pattern 3,4 vs. Pattern 0–2 (Ref.)	0.23	0.07–0.77	**0.017**	0.26	0.08–0.88	**0.030**

*Ref. refers to the stratification of variable as reference in the Cox hazard-proportional regression analysis*.

*HR, hazard ratio; 95% CI, 95% confidence interval; GGO, ground glass opacity; Three mixed signs, GGO, consolidation and linear opacity; Pattern 0, negative; Pattern 1, organizing pneumonia pattern; Pattern 2, progressive organizing pneumonia pattern; Pattern 4, diffuse alveolar damage pattern. The bold value refers to P < 0.05*.

## Discussion

By delineating the COVID-19 pneumonic CT patterns and their evolutional characteristics, this study aimed to determine their value in predicting adverse outcomes. Results indicated that CT Pattern 4 was associated with a higher rate of an adverse outcome after controlling for age; meanwhile, Pattern 3 and 4 showed more prevalence of pulmonary residuals on CT. Individual CT pattern for prognostic implication can be determined within 2 weeks after symptom onset due to the remarkable evolution of patterns before 2 weeks and subsequent stabilization or evolution without prognostic impacts.

Three kinds of phenotypes by characterizing the hypoxemia-related severity have been proposed to guide the respiratory treatment for COVID-19 (23–25). Among them, a two-phenotype of type L (low) and H (high) and a five-phenotype were defined to delineate the disease severity, mainly for hypoxemia state by clinical and/or imaging findings (23, 25). While, another three-phenotype stemmed from CT findings (multiple, focal, possibly overperfused GGO; inhomogeneously distributed atelectasis; a patchy, ARDS-like pattern) (24). These phenotype classifications could be supplement to Diagnosis and Treatment Protocol for Novel Coronavirus Pneumonia (Trial Version 7) (21). By comparison, our CT pattern categorization detailed the extent of lung injury in COVID-19. Among them, Pattern 2 to 4 showed compatible with CT signs of three-phenotype (24). Pattern 1 was found to be linked with a good prognosis as well as Pattern 0. This resembled the prior reports of H1N1 pneumonia (17). Pathologically, organization has been recognized as a common response in lung injury (15, 26). In this study, OP patterns accounted for 60% and the overall degree of lung injury especially for Pattern 2 was mild where reparative process and resolution of lesions seem to follow. Note that more prevalence of residuals may indicate a protracted disease course in Pattern 3. This may be related to older patients with comorbidity and decreased lymphocyte percentage. For Pattern 4, 85.7% cases had an adverse outcome. Pathologically, intraalveolar edema, fibrin, and variable cellular infiltrates with a hyaline membrane were observed in DAD (16, 27). It may be more severe disease, more prevalence of elevated creatine kinase, neutrophil percentage and C-reactive protein that led to the higher rate of adverse outcomes in Pattern 4. Previous studies have demonstrated the residual fibrosis in 38 and 85% of DAD survivals, which may be related to barotrauma due to mechanical ventilation or oxygen toxicity (28). Differently, fibrosis was not pathologically observed in COVID-19 death perhaps due to the short disease course of 15 days from onset to death (29). A long-term follow up of discharged DAD patients who survived after mechanical ventilation or continuous high-flow oxygen therapy would be required to further understand the sequelae.

Diverse evolutions with overlaps of progression and downgrading were found in Pattern 0–2 within 3 weeks and Pattern 3–4 within 2 weeks after onset. Most of them remained thereafter. It is noting that 28.6% of Pattern 1 progressed to Pattern 2 from 2 to 3 weeks. This evolution was consistent with prior report of acute and progressive characteristics of COVID-19 (11). In addition, this progression from Pattern 1 to 2 after 2 weeks may reflect the organization regarding lung repair and would have good prognosis (15). From the above, individual CT pattern for prognostic implication can be determined within 2 weeks after onset due to the remarkable evolution of patterns before 2 weeks and subsequent stabilization or evolution without prognostic impacts.

Univariate analysis indicated that age ≥65 years, presence of comorbidity (70% hypertension and diabetes mellitus), severe or critical illness, neutrophil percentage >75%, CT score ≥10, CT Pattern 4 were significantly related with adverse outcome. These findings echo the latest reports (7, 8). In details, a poor clinical outcome was associated with increased age (>65 years), presence of comorbidity as well as elevated levels of hypersensitive troponin I, leukocyte and neutrophil in COVID-19 patients (7–9). By multivariate analysis, only Pattern 4 was associated with an adverse outcome after controlling age. In our cohort, most of Pattern 4 cases were age ≥65 years (64.3%), presence of comorbidity (71.4%) and critical illness (57.1%). This may be the underlying reason regarding Pattern 4 as only significant factor in multivariate analysis. This further enhanced the potential role of CT pattern in predicting the risks of adverse outcomes in COVID-19.

As for pulmonary sequelae, CT Pattern 3 or 4 and elevated C-reactive protein were two independent factors associated with pulmonary residuals on CT. Pattern 3 and 4 showed more prevalence of pulmonary residuals than others. This may be linked with more severe CT findings of these cases with more number of lobe affected and CT scores. In concert with MERS studies that radiological sequelae can remain at least 1 year after infection (30), our study found similar but slighter residuals mainly presenting with linear opacities and/or a few consolidation and GGO. Beyond, elevated C-reactive protein may indicate the state of tissue injury and/or acute inflammation, which may suggest a risk indication of progression to a critical disease state (31). In this regard, elevated C-reactive protein may be predictive of radiological sequelae. Prior studies indicated that radiological sequelae from SARS and MERS may suggest the abnormal or repaired lung function (30, 32). Despite the slight residuals in COVID-19, a long-term follow-up is required to further trace the resolution and associations with lung function.

This study had some limitations. The first was the small sample, especially for those with adverse outcomes and/or with Pattern 4. A larger sample is required to further verify the findings regarding the risk factors affecting the adverse outcome and disease progression, as well as factors in relation to respiratory treatment strategy (e.g., non-invasive or mechanical ventilation). Besides, more clinical indicators such as body mass index would be gathered to explore the potential correlations with prognosis due to the prior report of obesity as risk factor of severe COVID-19 (33). Second, because discharged patients remained during the recovery and pulmonary CT residuals were unknown at the time of our analysis, a long-term follow-up is required to further trace the outcome of lesion absorption, as well as changes in lung functions. Third, despite of using a high-resolution CT protocol recommended by American College of Radiology (34), varying CT scanners may have potential impacts on CT pattern evaluation. A large sample from these CT scanners should be collected to first clarify the impacts and thereby facilitate the generalization of our findings. Forth, multicenter data collection may lead to selective bias of patients with various CT patterns. Although no significance in univariate analysis (see more in Supplement Material), potential impacts from varying hospital, epicenter vs. non-epicenter should be considered in further studies. Last, given the inadequate CT resource, an alternative pattern categorization by X-ray image and/or available quick-test laboratory indicators should be further explored.

In conclusion, CT pattern categorization of COVID-19 pneumonia based on chest CT within 2 weeks after symptom onset has prognostic significance. CT pattern 4 cases present high risk of admission to ICU, need for mechanical ventilation or death, while Pattern 3 and 4 signal likelihood of pulmonary residuals on CT. In this regard, when allocating medical resources, pattern 0–2 cases could be considered as mild group and then admitted to community hospital or mobile cabin hospital, while pattern 3 or 4 should be admitted to designate general hospital. These findings would help early prognostic stratification of COVID-19 and facilitate the decision making for treatment strategy and optimal use of healthcare resources.

## Data Availability Statement

The raw data supporting the conclusions of this article will be made available by the authors, without undue reservation.

## Ethics Statement

The studies involving human participants were reviewed and approved by The internal review board of the First Affiliated Hospital of Xi'an Jiaotong University. Written informed consent for participation was not required for this study in accordance with the national legislation and the institutional requirements.

## Author Contributions

CJ and JY contributed to the literature search. CT, YW, HZha, TL, ZLiu, ZJ, RL, ZW, FL, JZ, SC, YL, HL, ZLi, YL, HZho, XW, and ZR contributed to data collection and analysis. CJ, YW, and JY contributed to data interpretation. CJ, CT, CW, and JY contributed to writing of the manuscript. All authors contributed to the study conception, design, article, and approved the submitted version.

## Conflict of Interest

The authors declare that the research was conducted in the absence of any commercial or financial relationships that could be construed as a potential conflict of interest.

## References

[1] Fauci AS Lane HC Redfield RR. Covid-19 - navigating the uncharted. N Engl J Med. (2020) 382:1268–9. 10.1056/NEJMe2002387 32109011 PMC7121221

[2] WHO Coronavirus disease 2019. 2020. https://covid19.who.int/ (accessed May 29, 2020).

[3] Guan WJ Ni ZY Hu Y Liang WH Ou CQ He JX Clinical characteristics of coronavirus disease 2019 in China. N Engl J Med. (2020) 382:1708–20. 10.1056/NEJMoa2002032 32109013 PMC7092819

[4] Huang C Wang Y Li X Ren L Zhao J Hu Y. Clinical features of patients infected with 2019 novel coronavirus in Wuhan, China. Lancet. (2020) 395:497–506. 10.1016/S0140-6736(20)30183-5 31986264 PMC7159299

[5] Wang D Hu B Hu C Zhu F Liu X Zhang J. Clinical characteristics of 138 hospitalized patients with 2019 novel coronavirus-infected pneumonia in Wuhan, China. JAMA. (2020) 323:1061–9. 10.1001/jama.2020.1585 32031570 PMC7042881

[6] Pormohammad A Ghorbani S Baradaran B Khatam A Turner R Mansournia MA. Clinical Characteristics, Laboratory Findings, Radiographic Signs and Outcomes of 52,251 Patients With Confirmed COVID-19 Infection: A Systematic Review and Meta-Analysis. Preprints [Preprint]. (2020) Available online at: https://www.preprints.org/manuscript /202003.0252/v1. (assessed March 18, 2020) 10.1016/j.micpath.2020.104390 PMC7361116 32681968

[7] Zhou F Yu T Du R Fan G Liu Y Liu Z Clinical course and risk factors for mortality of adult inpatients with COVID-19 in Wuhan, China: a retrospective cohort study. Lancet. (2020) 395:1054–62. 10.1016/S0140-6736(20)30566-3 32171076 PMC7270627

[8] Zhao X Zhang B Li P Ma C Gu J Hou P Incidence, clinical characteristics and prognostic factor of patients with COVID-19: a systematic review and meta-analysis. medRxiv [Preprint]. (2020) 10.1101/2020.03.17.20037572

[9] Hu L Chen S Fu Y Gao Z Long H Ren H. Risk factors associated with clinical outcomes in 323 COVID-19 patients in Wuhan, China. Clin Infect Dis. (2020) 10.1093/cid/ciaa539. [Epub ahead of print]. 32361738 PMC7197620

[10] Hosseiny M Kooraki S Gholamrezanezhad A Reddy S Myers L. Radiology perspective of coronavirus xisease 2019 (COVID-19): lessons from severe acute respiratory syndrome and middle east respiratory syndrome. AJR Am J Roentgenol. (2020) 214:1078–82. 10.2214/AJR.20.22969 32108495

[11] Wang Y Dong C Hu Y Li C Ren Q Zhang X. Temporal changes of CT findings in 90 patients with COVID-19 pneumonia: a longitudinal study. Radiology. (2020) 296:E55–E64. 10.1148/radiol.2020200843 32191587 PMC7233482

[12] Shi H Han X Jiang N Cao Y Alwalid O Gu J. Radiological findings from 81 patients with COVID-19 pneumonia in Wuhan, China: a descriptive study. Lancet Infect Dis. (2020) 20:25–434. 10.1016/S1473-3099(20)30086-4 32105637 PMC7159053

[13] Wu J Wu X Zeng W Guo D Fang Z Chen L. Chest CT findings in patients with corona virus disease 2019 and its relationship with clinical features. Invest Radiol. (2020) 55:257–61. 10.1097/RLI.0000000000000670 32091414 PMC7147284

[14] Salehi S Abedi A Balakrishnan S. Coronavirus disease 2019 (COVID-19): a systematic review of imaging findings in 919 patients. Am J Roentgenol. (2020) 215:87–93. 10.2214/AJR.20.23034 32174129

[15] Kligerman SJ Franks TJ Galvin JR. From the radiologic pathology archives: organization and fibrosis as a response to lung injury in diffuse alveolar damage, organizing pneumonia, and acute fibrinous and organizing pneumonia. Radiographics. (2013) 33:1951–75. 10.1148/rg.337130057 24224590

[16] Xu Z Shi L Wang Y Zhang J Huang L Zhang C. Pathological findings of COVID-19 associated with acute respiratory distress syndrome. Lancet Respir Med. (2020) 8:420–2. 10.1016/S2213-2600(20)30076-X 32085846 PMC7164771

[17] Kang H Lee KS Jeong YJ Lee HY Kim KI Nam KJ. Computed tomography findings of influenza a (H1N1) pneumonia in adults: pattern analysis and prognostic comparisons. J Comput Assist Tomogr. (2012) 36:285–90. 10.1097/RCT.0b013e31825588e6 22592609

[18] Lee KS. Pneumonia associated with 2019 novel coronavirus: can computed tomographic findings help predict the prognosis of the disease? Korean J Radiol. (2020) 21:257–8. 10.3348/kjr.2020.0096 32056396 PMC7039716

[19] Paul NS Roberts H Butany J Chung TB Gold W. Radiologic pattern of disease in patients with severe acute respiratory syndrome: the Toronto experience. Radiographics. (2004) 24:553–63. 10.1148/rg.242035193 15026600

[20] WHO Clinical Management of Severe Acute Respiratory Infection When Novel Coronavirus (nCoV) Infection Is Suspected. (2020) Available online at: https://www.who.int/publications-detail /clinical-management-of-severe-acute-respiratory-infection-when-novel-coronavirus-(ncov)-infection-is-suspected (accessed March 15, 2020).

[21] National Health Commission of the People's Republic of China Diagnosis and Treatment of Pneumonia Caused by 2019-nCoV (version 7). Available online at: http://www.gov.cn/zhengce/zhengceku/ 2020-03/04/content_5486705.htm (accessed March 15, 2020).

[22] Chang YC Yu CJ Chang SC Galvin JR Liu HM Hsiao CH. Pulmonary sequelae in convalescent patients after severe acute respiratory syndrome: evaluation with thin-section CT. Radiology. (2005) 236:1067–75. 10.1148/radiol.2363040958 16055695

[23] Gattinoni L Chiumello D Caironi P Busana M Romitti F Brazzi L. COVID-19 pneumonia: different respiratorytreatment for different phenotypes? Intensive Care Med. (2020) 46:1099–102. 10.1007/s00134-020-06033-2 32291463 PMC7154064

[24] Robba C Battaglini D Ball L Patroniti N Loconte M Brunetti I. Distinct phenotypes require distinct respiratory management strategies in severe COVID-19. Respir Physiol Neurobiol. (2020) 279:103455. 10.1016/j.resp.2020.103455 32437877 PMC7211757

[25] Rello J Storti E Belliato M Ricardo S. Clinical phenotypes of SARS-CoV-2: Implications for clinicians and researchers. Eur Respir J. (2020) 55:2001028. 10.1183/13993003.01028-2020 32341111 PMC7236837

[26] Baque-Juston M Pellegrin A Leroy S Marquette CH Padovani B. Organizing pneumonia: what is it? A conceptual approach and pictorial review. Diagn Interv Imaging. (2014) 95:771–7. 10.1016/j.diii.2014.01.004 24559802

[27] Franquet T. Imaging of pulmonary viral pneumonia. Radiology. (2011) 260:18–39. 10.1148/radiol.11092149 21697307

[28] Nöbauer-Huhmann I-M Eibenberger K Schaefer-Prokop C Steltzer H Schlick W Strasser K. Changes in lung parenchyma after acute respiratory distress syndrome (ARDS): assessment with high-resolution computed tomography. Eur Radiol. (2001) 11:2436–43. 10.1007/s003300101103 11734936

[29] Liu Q Wang RS Qu GQ Wang YY Liu P Zhu YZ. Gross examination report of a COVID-19 death autopsy. Fa Yi Xue Za Zhi. (2020) 36:21–23. (In Chinese). 10.12116/j.issn.1004-5619.2020.01.005 32198987

[30] Park WB Jun KIl Kim G Choi JP Rhee JY Cheon S Correlation between pneumonia severity and pulmonary complications in Middle East respiratory syndrome. J Korean Med Sci. (2018) 33:e169 10.3346/jkms.2018.33.e169 29892209 PMC5990444

[31] Clyne B Olshaker JS. The C-reactive protein. J Emerg Med. (1999) 17:1019–25. 10.1016/S0736-4679(99)00135-3 10595891

[32] Antonio GE Wong KT Hui DSC Wu A Lee N Yuen EHY. Thin-section CT in patients with severe acute respiratory syndrome following hospital discharge: preliminary experience. Radiology. (2003) 228:810. 10.1148/radiol.2283030726 12805557

[33] Sattar N McInnes IB McMurray J. Obesity is a risk factor for severe COVID-19 infection: multiple potential mechanisms. Circulation. (2020) 142:4. 10.1161/CIRCULATIONAHA.120.047659 32320270

[34] American College of Radiology ACR-STR Practice Parameter for the Performance of High-Resolution Computed Tomography (HRCT) of the Lungs in Adults. Available online at: https://www.acr.org/-/media/ACR/Files/Practice-Parameters/HRCT-Lungs.pdf (accessed July 15, 2020).

