# Supplementary material for: A Pattern Categorization of CT Findings to Predict Outcome of COVID-19 Pneumonia
Source: Front Public Health. 2020 Sep 18;8:567672. doi: 10.3389/fpubh.2020.567672 (PMC7531052; doi:10.3389/fpubh.2020.567672)
Supplement: Supplementary file 1 [file Data_Sheet_1.docx]

Supplementary Material

# Characteristics of patients with COVID-19 in clinical outcome and post-discharge CT residuals

Supplementary Table 1 detailed the clinical characteristics, laboratory results and radiological findings of patients in clinical outcome and pulmonary CT residuals after discharge. In the full cohort, the mean age was 45.6 (SD, 16.4; range, 4-89) years and there was no gender difference (55 [58.5%] men, 39 [41.5%] women). Significant differences between discharge and adverse event were found in terms of age (*P*<0.001), disease severity (*P*<0.001), with underlying disease (*P*<0.001), symptoms of fatigue (P=0·018) and Chest congestion and/or shortness of breath (*P*=0.001), laboratory results (decreased lymphocyte percentage [*P*=0.036], decreased leukocyte count [*P*=0.037], elevated neutrophil percentage [*P*<0·001], elevated C-reactive protein [*P*<0·001]) and radiological findings (radiological pattern [*P*<0·001], proportion of GGO and consolidation [*P*<0.001], number of lobe involvement [*P*=0.005], CT score [*P*<0.001]).

For pulmonary sequelae, significant differences between complete absorption and residual lesions on CT were found in terms of age (*P*<0.001), elevated neutrophil percentage (*P*=0.032), elevated C-reactive protein (*P*<0.001) and radiological findings (radiological pattern [*P*=0.014], proportion of GGO, consolidation and linear opacity [*P*=0.043], number of lobe involvement [*P*=0.009], CT score [*P*=0.009]).

# Reliability for inter-observer agreement in CT pattern categorization

Given the ordinal characteristic of CT pattern categorization, the Kendall's tau-b correlation coefficient was used to test the reliability for inter-observer agreement. Results indicated that Kendall's tau-b correlation coefficient was 0·865 (P<0·001) suggesting a good performance of inter-observer agreement in pattern categorization.

# Cutoff CT score for pattern discrimination

Receiver operating characteristic curve analysis was used to estimate the cutoff value of CT score in discriminations of Pattern 2 vs. 3 and Pattern 3 vs. 4 due to their similar CT signs. Results indicated that cutoff CT score of 6 enabled the discrimination of Pattern 2 vs. 3 (area under curve, 0·77; P<0·001), while cutoff value of 10 identified Pattern 4 from Pattern 3 (area under curve, 0·94; P<0·001) (Supplementary Figure 1).

**Supplementary Figure 1.** Plots of receiver operating characteristic (ROC) curve for pattern differentiations. (A) Pattern 2 vs. Pattern 3, and (B) Pattern 3 vs. Pattern 4. AUC = area under ROC curve.

# Impacts of varying hospital on clinical outcome

The univariate Cox proportional hazards regression indicated that varying hospital was not significantly related with an adverse outcome (Hazard ratio [HR] 1.25, 95%CI 0.93-1.67, P=0.146).

**Supplementary Table 1** Characteristics of patients with COVID-19 pneumonia in primary clinical outcome and pulmonary sequelae on CT after discharge.

| **Characteristic** | **All**  **(*n*=94)** | **Primary clinical outcome** | | |  | **Pulmonary sequelae on CT after discharge^b^** | | |
| --- | --- | --- | --- | --- | --- | --- | --- | --- |
|  |  | **Discharge**  **(*n*=81)** | **Adverse event**  **(*n*=13)** | ***P* value** |  | **Complete absorption (*n*=31)** | **Residual lesion (*n*=50)** | ***P* value** |
| Age (yr)^a^ | 45.6±16.4 | 42.2±14.0 | 66.9±14.6 | **<0**.**001** |  | 35.2±13.1 | 46.5±12.8 | **<0**.**001** |
| Male sex | 55(58.5) | 47(58.0) | 8(61.5) | 0.811 |  | 13(41.9) | 21(42.0) | 0.995 |
| Disease severity |  |  |  | **<0**.**001** |  |  |  | 0.858 |
| Mild | 78(83.0) | 78(96.3) | 0 |  |  | 30(96.8) | 48(96.0) |  |
| Severe | 8(8.5) | 3(3.7) | 5(38.5) |  |  | 1(3.2) | 2(4.0) |  |
| Critical illness | 8(8.5) | 0 | 8(61.5) |  |  | 0 | 0 |  |
| Comorbidity | 32(34.0) | 22(27.2) | 10(76.9) | **<0**.**001** |  | 5(16.1) | 17(34.0) | 0.079 |
| Clinical symptom at admission |  |  |  |  |  |  |  |  |
| Fever | 81(86.2) | 68(84.0) | 13(100) | 0.120 |  | 23(74.2) | 48(96.0) | 0.060 |
| Fatigue | 20(21.3) | 14(17.3) | 6(46.2) | **0**.**018** |  | 5(16.1) | 9(18.0) | 0.829 |
| Pharyngalgia | 15(16.0) | 12(14.8) | 3(23.1) | 0.450 |  | 4(12.9) | 8(16.0) | 0.703 |
| Headache | 5(5.3) | 0 | 5(38.5) | 0.357 |  | 1(3.2) | 4(8.0) | 0.386 |
| Cough | 49(52.1) | 41(50.6) | 8(61.5) | 0.464 |  | 14(45.2) | 27(54.0) | 0.439 |
| Expectoration | 24(25.5) | 19(23.5) | 5(38.5) | 0.249 |  | 4(12.9) | 15(30.0) | 0.078 |
| Chest congestion and/or shortness of breath | 14(14.9) | 8(9.9) | 6(46.2) | **0**.**001** |  | 2(6.5) | 6(12.0) | 0.416 |
| Muscle soreness | 7(7.4) | 6(7.4) | 1(7.7) | 0.971 |  | 2(6.5) | 4(8.0) | 0.796 |
| Nausea and vomiting | 1(1.1) | 1(1.2) | 0 | 0.687 |  | 0 | 1(2.0) | 0.428 |
| Diarrhea | 3(3.2) | 2(2.5) | 1(7.7) | 0.320 |  | 0 | 2(4.0) | 0.260 |
| No symptom | 4(4.3) | 4(4.9) | 0 | 0.413 |  | 2(6.5) | 2(4.0) | 0.621 |
| Laboratory test at admission |  |  |  |  |  |  |  |  |
| Lymphocyte percentage (%) |  |  |  | **0**.**036** |  |  |  | 0.069 |
| <20 | 40(42.6) | 31(38.3) | 9(69.2) |  |  | 8(25.8) | 23(46.0) |  |
| ≥20 | 54(57.4) | 50(61.7) | 4(30.8) |  |  | 23(74.2) | 27(54.0) |  |
| Monocyte percentage (%) |  |  |  | 0.130 |  |  |  | 0.185 |
| >10 | 23(24.5) | 22(27·2) | 1(7.7) |  |  | 11(35.5) | 11(22.0) |  |
| ≤10 | 71(75.5) | 59(72·8) | 12(92.3) |  |  | 20(64.5) | 39(78.0) |  |
| Leukocyte count (10^9^/L) |  |  |  | **0**.**037** |  |  |  | 0.288 |
| <3.5 | 21(22.3) | 21(25.9) | 0 |  |  | 6(19.4) | 15(30.0) |  |
| ≥3.5 | 73(77.7) | 60(74.1) | 13(100) |  |  | 25(80.6) | 35(70.0) |  |
| Alanine Aminotransferase (U/L) |  |  |  | 0.614 |  |  |  | 0.412 |
| >50 | 17(18.1) | 14(17.3) | 3(23.1) |  |  | 4(12.9) | 10(20.0) |  |
| ≤50 | 77(81.9) | 67(82.7) | 10(76.9) |  |  | 27(87.1) | 40(80.0) |  |
| Aspartate Aminotransferase (U/L) |  |  |  | 0.103 |  |  |  | 0.663 |
| >40 | 20(21.3) | 15(18.5) | 5(38.5) |  |  | 5(16.1) | 10(20.0) |  |
| ≤40 | 74(78.7) | 66(81.5) | 8(61.5) |  |  | 26(83.9) | 40(80.0) |  |
| Creatine kinase (U/L) |  |  |  | **0.017** |  |  |  | 0.581 |
| >310 | 10(10.6) | 7(8.6) | 3(23.1) |  |  | 2(6.5) | 5(10.0) |  |
| ≤310 | 84(89.4) | 74(91.4) | 10(76.9) |  |  | 29(93.5) | 45(90.0) |  |
| Neutrophil percentage (%) |  |  |  | **<0**.**001** |  |  |  | **0**.**032** |
| >75 | 30(31.9) | 18(22.2) | 12(92·3) |  |  | 3(9.7) | 15(30.0) |  |
| ≤75 | 64(68.1) | 63(77·8) | 1(7.7) |  |  | 28(90.3) | 35(70.0) |  |
| C-reactive protein (mg/L) |  |  |  | **<0**.**001** |  |  |  | **<0**.**001** |
| >10 | 52(55.3) | 39(48.1) | 13(100) |  |  | 7(22.6) | 32(64.0) |  |
| ≤10 | 42(44.7) | 42(51.9) | 0 |  |  | 24(77.4) | 18(36.0) |  |
| Hemoglobin (g/L) |  |  |  | 0.372 |  |  |  | 0.140 |
| <130 | 14(14.9) | 11(13.6) | 3(23.1) |  |  | 2(6.5) | 9(18.0) |  |
| ≥130 | 80(85.1) | 70(86.4) | 10(76.9) |  |  | 29(93.5) | 41(82.0) |  |
| CT findings within 2 weeks after onset |  |  |  |  |  |  |  |  |
| Radiological pattern |  |  |  | **<0**.**001** |  |  |  | **0**.**014** |
| Pattern 0 | 3(3.2) | 3(3.7) | 0 |  |  | 1(3.2) | 2(4.0) |  |
| Pattern 1 | 15(16.0) | 15(18.5) | 0 |  |  | 10(32.3) | 5(10.0) |  |
| Pattern 2 | 46(48.9) | 46(56.8) | 0 |  |  | 17(54.8) | 22(44.0) |  |
| Pattern 3 | 16(17.0) | 15(18.5) | 1(7.7) |  |  | 3(9.7) | 19(38·0) |  |
| Pattern 4 | 14(14.9) | 2(2.5) | 12(92.3) |  |  | 0 | 2(4.0) |  |
| CT signs |  |  |  |  |  |  |  |  |
| GGO only | 6(6.4) | 5(6.2) | 1(7.7) | 0.835 |  | 3(9.7) | 2(4.0) | 0.302 |
| Consolidation | 8(8.5) | 8(9.9) | 0 | 0.236 |  | 5(16.1) | 3(6.0) | 0.138 |
| GGO and consolidation | 26(27.7) | 17(21.0) | 9(69.2) | **<0**.**001** |  | 8(25.8) | 9(18.0) | 0.402 |
| Linear opacity | 0 | 0 | 0 | -- |  | 0 | 0 | -- |
| GGO and linear opacity | 5(5.3) | 5(6.2) | 0 | 0.357 |  | 2(6.5) | 3(6.0) | 0.935 |
| Consolidation and linear opacity | 4(4.3) | 4(4.9) | 0 | 0.413 |  | 1(3.2) | 3(6.0) | 0.575 |
| Three signs | 38(40.4) | 35(43.2) | 3(23.1) | 0.170 |  | 9(29.0) | 26(52.0) | **0**.**043** |
| Lobe involvement |  |  |  | **0**.**005** |  |  |  | **0**.**009** |
| Number of lobe affected >3 | 53(56.4) | 41(50.6) | 12(92.3) |  |  | 10(32.3) | 31(62.0) |  |
| Number of lobe affected ≤3 | 41(43·6) | 40(49.4) | 1(7.7) |  |  | 21(67.7) | 19(38.0) |  |
| CT severity score^a^ | 5.8±4.4 | 4.6±3.1 | 13.3±3.6 | **<0**.**001** |  | 3.5±2.9 | 5.3±3.1 | **0**.**009** |

Note: Unless otherwise indicated, data are reported as the number of patients, with percentages in parentheses.

a, data are reported as the mean±standard derivation.

b, pulmonary sequelae on CT refer to the resolution status of lesions at the first post-discharge follow-up visit with a median of 37 (range, 14-58) days after symptom onset.

Abbreviations: GGO = ground glass opacity; Three signs = GGO, consolidation and linear opacity.
